# 兔肺VX2鳞癌移植瘤射频消融后残存肿瘤ERCC1表达的变化

**DOI:** 10.3779/j.issn.1009-3419.2013.12.01

**Published:** 2013-12-20

**Authors:** 连君 马, 乃康 周, 彦君 祁, 慧峰 刘, 亚超 赵, 梦利 郑

**Affiliations:** 1 100853 北京，解放军总医院胸外科 Department of Thoracic Surgery, Chinese PLA General Hospital, Beijing 100853, China; 2 100091 北京，解放军309医院胸外科 Department of Thoracic Surgery, Chinese PLA 309^th^ Hospital, Beijing 100091, China

**Keywords:** 射频消融, VX2鳞癌, 残存肿瘤, ERCC1, 肺, Radiofrequency ablation, VX2 squamous carcinoma, Residual tumor, ERCC1, Lung

## Abstract

**背景与目的:**

残存肿瘤是影响射频消融（radiofrequency ablation, RFA）治疗肺恶性肿瘤效果的重要因素，联合铂类药物化疗是减少残存肿瘤的重要手段之一。核苷酸切除修复交叉互补基因1（excision repair cross-complementation group 1, ERCC1）的表达水平是影响铂类药物化疗效果的重要因素之一。RFA治疗后残存肿瘤会发生一些生物学特性变化，但有关ERCC1表达变化的研究尚无报道。本研究旨在探讨RFA治疗后兔肺内残存VX2鳞癌细胞ERCC1表达水平的变化。

**方法:**

应用组织块悬液注射法建立兔VX2鳞癌肺内移植瘤模型。58只荷瘤新西兰白兔随机分为对照组（*n*=10）和RFA组（*n*=48）。在RFA治疗时，通过控制电极展开范围、输出功率、治疗时间的方法，造成肿瘤残存。应用免疫组织化学方法检测残存肿瘤细胞在不同时间点ERCC1表达的阳性率。

**结果:**

RFA组残存肿瘤组织ERCC1表达阳性率在1 d-5 d呈一过性升高（53.7%±1.6% & 32.9%±2.5%），5 d后恢复至对照组水平。

**结论:**

因在RFA治疗后1 d-5 d内残存肿瘤细胞ERCC1表达增高，在此期间给予铂类药物化疗可能效果不佳。

## Abstract (en)

**Background and objective:**

Residual carcinoma cells play an important role in the result of radiofrequency ablation (RFA) of pulmonary malignancies, and Platinum-based adjuvant chemotherapy is one of the important treatment regimen to reduce residual carcinoma cells after RFA. ERCC1 (excision repair cross-complementation group 1) is an important factor affecting Platinum-based chemotherapy effects. Residual carcinoma cells exhibit some changes of their features after RFA; however, there is no report about the change of their ERCC1 expression by now. This study focused on the change of ERCC1 expression in residual VX2 squamous carcinoma cells in rabbit lung after RFA.

**Methods:**

The model of VX2 squamous carcinoma in rabbit lung was established by injection of tissue block suspension. Fifty-eight New Zealand White rabbits with VX2 squamous carcinoma were randomly devided into the control group (*n*=10) and the RFA group (*n*=48). During the RFA procedure in these models, residual carcinoma cells were achieved by controlling the range of electrode expanding, power output and treatment time. At different points of time, the positive rates of ERCC1 expression in residual carcinoma were detected by immunohistochemistry.

**Results:**

Comparing with the control group, the positive rate of ERCC1 expression in residual carcinoma in RFA group increases transiently within 1 d to 5 d (53.7%±1.6% & 32.9%±2.5%), and 5 d later, it decline to the level of the control group.

**Conclusion:**

The ERCC1 expression of residual pulmonary carcinoma increase within 5 d after RFA. Thus platinum-based adjuvant chemotherapy may be ineffective in this period.

射频消融（radiofrequency ablation, RAF）是通过射频电流引起离子振荡和极性生物大分子极性转换，造成摩擦，使靶区组织升温，导致凝固性坏死的一种局部热损毁手段，具有操作方便、创伤小、恢复快的特点^[1]^。在胸部恶性肿瘤领域，RFA主要用于不能耐受手术的早期非小细胞肺癌（non-small cell lung cancer, NSCLC）和肺内转移肿瘤的治疗^[2]^。随着RFA应用的普及，人们发现残存肿瘤细胞是影响其远期疗效的最主要因素^[3, 4]^，RFA联合铂类药物化疗是减少术后肿瘤细胞残存的有效手段之一，但在具体的结合模式方面，尚缺乏统一认识^[5, 6]^。近年来，人们对RFA治疗后残存肿瘤细胞的一些生物学特性改变进行了深入研究^[7-10]^。ERCC1表达情况常用作肺癌对铂类化疗药物敏感性的预测指标，也用作肺癌患者预后指标^[11]^，目前尚无肺肿瘤RFA治疗后残存肿瘤细胞ERCC1表达情况的研究报道。兔VX2肿瘤源于Shop病毒诱导的兔乳头状瘤衍生鳞癌，有高侵袭性和高转移性，可在多种兔子、多种脏器建立移植瘤模型^[12]^。为此，我们采用免疫组织化学方法对RFA治疗后兔肺内残存VX2鳞癌细胞ERCC1表达情况进行了研究。

## 1 材料与方法

### 1.1 荷瘤动物模型制备和分组

70只健康纯种新西兰白兔，3月-4月龄，体重2.0 kg-2.5 kg，雌雄不限（由解放军总医院实验动物中心提供）；肌肉注射3%戊巴比妥钠（1 mL/kg）麻醉后，右胸壁脱毛，采用组织块悬液肺内注射法^[12]^建立VX2鳞癌肺内移植瘤模型（VX2鳞癌细胞株由解放军总医院肿瘤中心提供）；然后随机分为对照组10只，RFA组60只。RFA组再按时间分为0 d（即时）、1 d、3 d、5 d、7 d、14 d共6个组，每组10只。

### 1.2 RFA治疗和取材

待新西兰白兔肺内VX2鳞癌生长至最大径超过10 mm后进行RFA治疗。以前述方法麻醉，3 mm层厚连续胸部CT扫描定位，置入10针集束电极，确认其尖端位于肿瘤中心后，控制电极展开直径不超过8 mm。开启RF2000型射频机（Radiotherapeutic^TM^公司生产），起始输出功率为30 W，每分钟升高10 W，最高输出功率60 W，治疗4 min，退出电极。CT扫描有无血、气胸发生，并及时进行胸腔穿刺治疗。术后常规饲养，并肌肉注射青霉素160万单位/天（0 d-3 d）。各RFA组实验动物按相应时间点处死，取凝固性坏死边界内外各3 mm的肿瘤组织，4%中性甲醛固定后，常规制备石蜡切片，HE染色观察，以同时含有凝固性坏死肿瘤和残存肿瘤的组织为合格标本，否则为不合格标本。对照组实验动物仅置入并展开电极，不进行RFA治疗即处死，取周边的肿瘤组织作为研究标本。

### 1.3 免疫组织化学染色

对照组和RFA组合格标本的石蜡块连续4 μm厚切片，按Envision法进行免疫组织化学染色。即用型鼠抗兔ERCC1单克隆抗体（克隆号：8F1）及免疫组织化学检测试剂盒购自福州迈新生物技术开发有限公司，实验步骤按试剂盒说明书进行。以提供的阳性切片在同一条件下染色作为阳性对照，用PBS替代一抗作为阴性对照。利用Image-Pro Plus 6.0软件进行图像分析，以细胞核内发现棕黄色颗粒为阳性，每例标本在400倍光镜下选取5个有代表性的区域进行计数，共计数500个-1, 500个肿瘤细胞，计算阳性细胞百分率。

### 1.4 统计学处理

采用SPSS 13.0软件进行数据分析，肿瘤细胞ERCC1表达的阳性百分率采用Mean±SD表示，各RFA组均同对照组进行比较，采用*t*检验，*P* < 0.05为差异有统计学意义，检验和*P*值均为双侧。

## 2 结果

### 2.1 合格标本数量

RFA组共获得48例合格标本。在12例不合格标本中，7例因为实验动物死于RFA并发症未达相应时间点而剔除（大量气胸2例，大量血胸1例，广泛肺不张4例）；其余5例因HE染色未见残存肿瘤细胞，不符合本实验要求而剔除。

### 2.2 残存肿瘤的分布

对RFA组合格标本HE染色观察发现，绝大多数残存VX2鳞癌细胞位于凝固性坏死灶周边，成片存在（图 1）；在凝固性坏死灶内较大血管周围偶见残存肿瘤细胞；凝固性坏死灶的其它区域未见残存肿瘤细胞。

**1:**
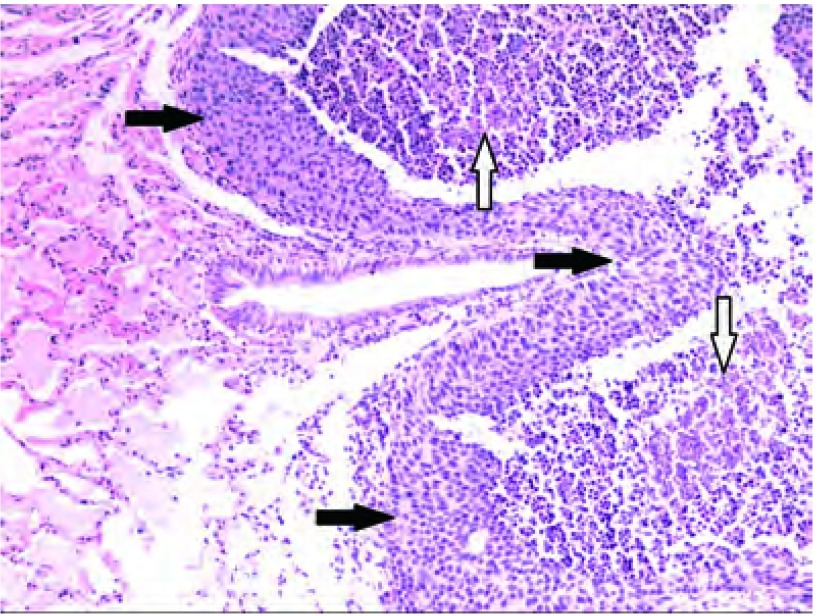
兔肺内VX2鳞癌RFA治疗后的残存肿瘤细胞和凝固性坏死肿瘤细胞（HE, ×100）。白色箭头：凝固性坏死的肿瘤细胞；黑色箭头：残存肿瘤细胞。 Residual cells and coagulation necrosis cells of VX2 squamous carcinoma in rabbit lung after RFA (HE, ×100). Black arrows: residual cells; White arrows: coagulation necrosis cells. RFA: radiofrequency ablation.

### 2.3 ERCC1免疫组织化学染色结果

对照组肿瘤细胞以及RFA组残存肿瘤细胞均见ERCC1表达（图 2），而凝固性坏死的肿瘤细胞未见ERCC1表达。对照组（*n*=10）肿瘤细胞ERCC1表达阳性率为32.9%±2.5%；1 d（*n*=7）、3 d（*n*=6）、5 d（*n*=8）组残存肿瘤细胞的ERCC1表达阳性率分别为50.7%±1.4%、53.7%±1.6%和36.9%±2.5%，明显高于对照组（*P* < 0.05），尤其以3 d组最高（*t*=18.23, *P* < 0.001）；而0 d（*n*=9）、7 d（*n*=10）、14 d（*n*=8）组的残存肿瘤细胞ERCC1表达阳性率分别为31%±2.1%、33.7%±1.4%和32.8%±1.4%，同对照组比较无明显差异（*P*>0.05）。

**2:**
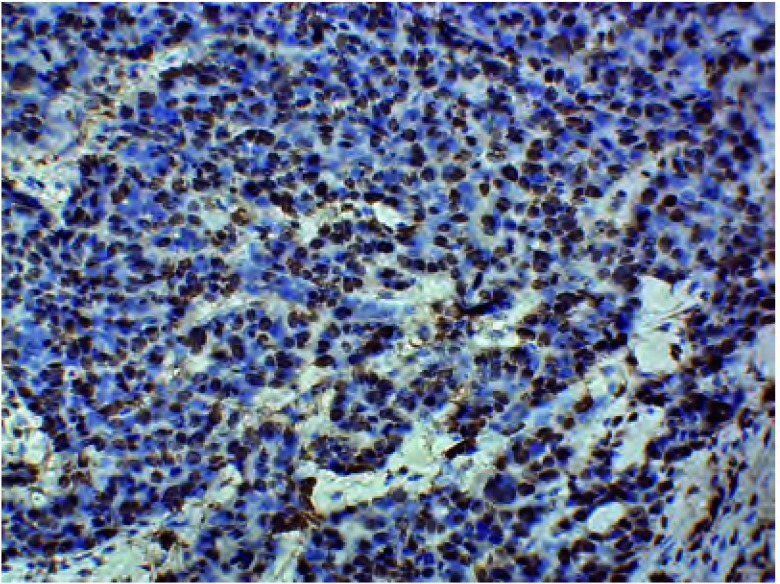
RFA治疗后兔肺内残存VX2鳞癌细胞ERCC1的表达(IHC, ×400) ERCC1 expression of in residual VX2 squamous carcinoma cells in rabbit lung after RFA (IHC, ×400)

## 3 讨论

肺内肿瘤RFA治疗效果同肺的组织学特点密切相关。由于大量气体的存在，导致肺内电传导和热传导的效应远低于其它实质性脏器；另外，在肺肿瘤周边持续存在的血流、气流又造成明显的“散热效应”。这两种因素导致RFA靶区周边以及靶区内部较大血管周围容易形成肿瘤残存^[13]^。虽然，近年通过改良电极等手段扩大了消融范围，但残存肿瘤问题仍没有得到根本解决^[4]^，肺内恶性肿瘤RFA治疗后残存肿瘤发生率为12%-40%^[1]^。在本研究中，我们通过控制电极展开范围、输出功率和治疗时间的方法，建立了兔VX2鳞癌肺移植瘤RFA治疗后的残存肿瘤模型。通过HE染色观察发现，这种模型的残存肿瘤细胞大多位于凝固性坏死灶的周围，在较大血管周围发现少量的残存肿瘤细胞，这一结果同其它文献报道^[1, 4, 13, 14]^相吻合。采用这种方法建立的兔肺内肿瘤RFA后残存肿瘤模型，方法简单、安全，成功率高，能够很好的模拟RFA后肺内残存肿瘤的实际状态，为相关研究提供了合适的平台。

RFA治疗后残存肿瘤对于肺肿瘤患者远期生存率的影响很大。von Meyenfeldt等^[14]^报道肺转移瘤患者RFA治疗后有肿瘤残存者3年生存率为49%，而无肿瘤残存者3年生存率为79%。近年来，人们尝试联合含铂类药物化疗，以减少RFA治疗后残存肿瘤细胞，提高治疗效果，但是在铂类药物给药时机方面尚缺乏统一的认识^[5, 6]^。铂类药物通过造成DNA链间交联引发的一系列反应最终导致细胞死亡，这种DNA链间交联可以被细胞内的核酸切除修复（nucleotide excision repair, NER）机制清除，从而修复铂类药物造成的DNA损伤，消弱其细胞毒性，导致耐药。ERCC1是NER的限速酶，是公认的反映NER功能的生物标记物，临床上常作为判断肿瘤细胞对铂类药物敏感性的标志物使用^[11]^。Olaussen等^[15]^经对761例NSCLC手术标本的ERCC1表达分析，发现术后含铂类药物化疗能延长ERCC1低表达患者的生存，但对于ERCC1高表达的患者无益处；并且ERCC1高表达可能意味着对铂类耐药。另一方面，ERCC1表达情况与NSCLC患者的预后也密切相关^[16, 17]^，ERCC1高表达的患者预后好于ERCC1低表达的患者，其原因是ERCC1低表达提示NER功能低下，容易导致DNA损伤积累增加，故患者预后差；而ERCC1高表达则相反。近期的一些研究^[7-10]^发现RFA治疗后残存肿瘤细胞的增殖、侵袭以及转移能力发生了改变，但关于ERCC1表达变化的研究尚未见报道。本研究采用免疫组织化学方法检测了RFA治疗后兔肺内VX2鳞癌残存肿瘤细胞ERCC1表达，结果显示在RFA治疗后1 d-5 d内残存肿瘤细胞ERCC1表达明显高于对照组水平，5 d后降至对照组水平。这一现象提示肺内残存的VX2鳞癌细胞受亚致死性热损伤后NER功能增强，这可能是一种自身保护性反应^[18]^，但这种反应的可能导致铂类药物的细胞毒性下降，影响化疗效果。结合Olaussen等^[15]^的研究结果，我们认为在RFA治疗后5 d内给予铂类药物化疗，可能效果不佳。另外，因在RFA治疗5 d后兔肺内残存VX2鳞癌细胞ERCC1表达阳性率降至对照组水平，故从判断预后角度看，无明显影响。

本研究仅是在动物模型上进行的肺内肿瘤RFA治疗后残存肿瘤ERCC1表达改变的初步探索，对于判断RFA治疗后铂类药物化疗时机还存在一定的局限性和不足之处。肺内肿瘤有多种细胞类型，而本研究仅为一种肿瘤细胞的实验结果，故有待其它类型肿瘤细胞表达数据及临床治疗研究的佐证。另外，影响铂类药物化疗效果的因素还有很多，比如RFA治疗后残存肿瘤细胞的细胞周期改变、肿瘤局部血液供应改变等，故在这些方面还需要深入研究。
